# Class I Myosins, molecular motors involved in cell migration and cancer

**DOI:** 10.1080/19336918.2021.2020705

**Published:** 2022-01-03

**Authors:** Juan D. Diaz-Valencia, Laura A. Estrada-Abreo, Leonor Rodríguez-Cruz, Alfonso R. Salgado-Aguayo, Genaro Patiño-López

**Affiliations:** a Immunology and Proteomics Laboratory, Children’s Hospital of Mexico, Mexico City, Mexico; b Cell Biology and Flow Cytometry Laboratory, Metropolitan Autonomous University, México City, Mexico; c Rheumatic Diseases Laboratory, National Institute of Respiratory Diseases “Ismael Cosío Villegas”, Mexico City, Mexico

**Keywords:** Class I Myosin, cancer, leukemia, cell migration, tissue infiltration, metastasis

## Abstract

Class I Myosins are a subfamily of motor proteins with ATPase activity and a characteristic structure conserved in all myosins: A N-Terminal Motor Domain, a central Neck and a C terminal Tail domain. Humans have eight genes for these myosins. Class I Myosins have different functions: regulate membrane tension, participate in endocytosis, exocytosis, intracellular trafficking and cell migration. Cell migration is influenced by many cellular components including motor proteins, like myosins. Recently has been reported that changes in myosin expression have an impact on the migration of cancer cells, the formation of infiltrates and metastasis. We propose that class I myosins might be potential markers for future diagnostic, prognostic or even as therapeutic targets in leukemia and other cancers.

**Abbreviations:** Myo1g: Myosin 1g; ALL: Acute Lymphoblastic Leukemia, TH1: Tail Homology 1; TH2: Tail Homology 2; TH3: Tail Homology 3.

## Introduction

Myosins are motor proteins with different functions within the cell, such as regulation of cell morphology, signaling; or trafficking of organelles, but their main function is to generate force and movement [1]. Eukaryotes have 38 classes of myosins [2]. These proteins have been classified in two groups, conventional and non-conventional, as a result of the genetic diversification in their structure. The most studied myosins are class II (conventional myosins), which are encoded by 15 genes in humans. Only recently myosins have been implicated in tumorigenesis and cancer progression, for recent reviews see [3–5]. Our research group has focused on the study of class I myosins a subfamily of eight members of the unconventional myosins that control plasma membrane tension [6,7] In this paper, we aim to highlight how class I myosins could contribute to cancer development and/or severity with special focus of Myo1g in Leukemia.

### Class I myosins, motor proteins that regulate vital processes in the cell

**Figure 1.**
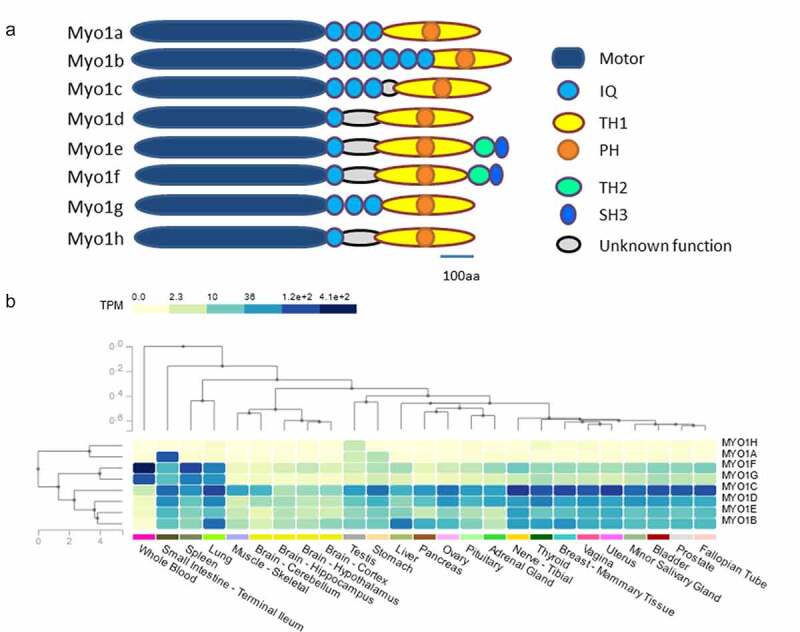
**Class I myosin family structural organization and tissue expression**. a) Schematic representation of the class I myosin family highlighting the main structural domains, Motor, Neck and Tail. b) Class I myosin gene expression heatmaps constructed with GTEX v8 (24 tissues), note the highly restricted expression pattern of Myo1h, Myo1a and Myo1g and the widely distributed expression of the remaining five class I myosins.

Class I myosins are a family of non-processive molecular motors attached to actin filaments; the interaction between these proteins generates movement in the cell [5,8,9]. In human, there are eight genes for these motor proteins: Myo1a, Myo1b, Myo1c, Myo1d, Myo1e, Myo1f, Myo1g, and Myo1h [8,9]. Class I Myosins are structurally composed of a single heavy chain of 110–140 KDa, divided into three regions: head, neck, and tail [10,11]. The motor or head domain contains the actin-binding site; this region is responsible for the ATP hydrolysis and movement along actin filaments. The Neck is characterized by one or several IQ motifs, these are composed of approximately 29 amino acid residues with the core sequence IQXXXRGXXXRXY (I–isoleucine, Q-glutamine, R-arginine, G-glycine, Y-tyrosine, and X-any other amino acid), each motif provides a binding site for calmodulin, a calcium-binding cytosolic protein that is the main light chain for this family of myosins [12,13]. The last domain of class I myosins is the tail; this domain is divided into TH (tail homology) regions, designated as TH 1, TH 2, and TH 3. Short tail myosins contain a unique TH1 region, whereas long tail myosins contain up to three TH regions [14]. The tail interacts with phospholipids and membrane proteins, facilitating direct binding to cell membranes [15,16] (Figure 1a), some of the class I Myosins present restricted tissue expression like Myo1a, Myo1h and Myo1g while the rest are more widely distributed (Figure 1b). The eight class I myosins play a role in different cellular processes, such as intracellular transport, formation of cell surface projections, regulation of endocytosis, exocytosis and phagocytosis, regulation of the cytoskeleton plasticity, membrane tension and cell migration [8,9,17,18].

The functions of these motor proteins have been studied in cells of the immune system (T and B lymphocytes), macrophages, enterocytes, kidney cells, oligodendrocytes, liver cells, and ear sensory cells [6,19–25], and only recently was reported that changes in expression and mutations in class I myosins contribute to diseases such as cancer [26–29].

### Cancer, migration, and metastasis

Cancer is a collection of chronic diseases characterized by many different genetic and molecular alterations, which ultimately result in uncontrolled cellular proliferation, resistance to apoptosis, evasion of growth suppressors, induction of angiogenesis, enabling replicative immortality and activating invasion and metastasis among others such as: genome Instability and mutation, deregulation of cellular energetics, tumor promoting inflammation and avoiding Immune destruction [30–32]. Abnormal cells grow invasively, and they spread to tissues, resulting in a lack of control in the normal physiology of the organism. Any organ or tissue might be affected, which is why there are more than 100 cancer types known. Worldwide, breast, cervix, colorectal, lung, stomach, prostate, and liver tumors are the most common forms of cancer in adults [33,34]. In children and teenagers, the main cause of morbidity and mortality is Acute Lymphoblastic Leukemia (ALL) [35,36]. This neoplasm represents 31% of the cancer diagnosed in children under 15 years old, with 30 to 40 cases per million per year. Disadvantaged prognosis or higher risk is associated with patients with a leukocyte count greater than 50,000, younger than one year old and older than ten years old, presence of cytogenetic alterations, failure to induce remission, early relapse to bone marrow and very importantly Testis or Central Nervous System infiltration.

Cancer cell dissemination causes tissue infiltrations in hematopoietic cancers and metastases in solid tumors. Invasion or metastasis is divided into the following stages: local invasion, migration, intravasation, extravasation, and metastasis [32,37–39]. During the first stage, cells invade its tissue of origin, making changes in the basement membrane to directly invade into the stroma. They subsequently migrate to enter the lumen of a blood or lymphatic vessel, diffusion through the circulation allows the metastatic cells to disperse. This intravasation process is facilitated by molecular changes that increase and promote the ability of cancer cells to cross through the endothelial barrier. Once the cancerous cells reach the venous or arterial circulation, they must survive the tensions of the hemodynamic environment so that they can settle to distant organs. Tumor cells survive in the microenvironment of an unusual tissue, which have an environment that is not always optimal for them to proliferate and form the metastatic colony [38,39].

**Figure 2.**
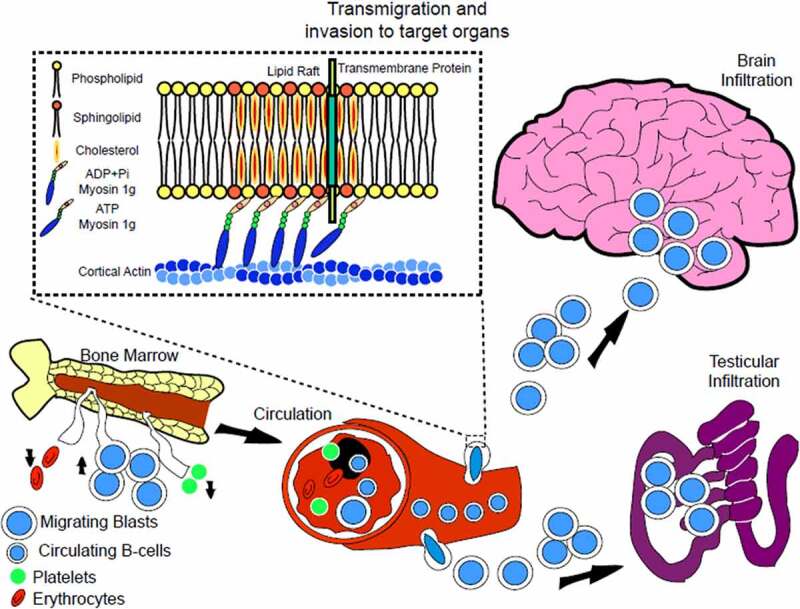
**Leukemic cells produce extramedullary infiltrates in Central Nervous System and Testis**. Patients with Acute Lymphoblastic Leukemia that present infiltrations at diagnosis are classified as high-risk patients. The severity of the patient’s condition is related to the presence of infiltrates, neutropenia, thrombocytopenia, hemorrhages, and acute anemia; Thrombocytopenia results in a decrease in blood volume due to frequent bleedings in patients. The decrease in blood volume creates a pathophysiological environment conducive to the formation of infiltrates and this combined with intrinsically improved cell migration characteristics of the leukemic cells due to over expression of Myo1g that increases plasma membrane tension and upregulation of adhesion molecules such as CD44 (dotted square) will result in infiltration into the CNS and testis in high-risk patients.

To date there is a scarce understanding of the molecular mechanisms of ALL infiltration to the CNS and testis, one pathway involves the use of Neural stem/Precursor cells (NSPC) embryonic pathfinding mechanism by ALL cells as a means to get across the vascular channels connecting bone marrow and meninges, the leading places of CNS ALL disease in humans [40]. Although there might be other unrecognized mechanisms and pathways that allow ALL cells to reach their target sites. Since high-risk patients with ALL present common extramedullary infiltrations [41], we propose that those infiltrations are, in part, an outcome of the combination of an improved leukemic cell migration and bleeding, abnormal loss of blood volume may allow the leukemic cells to establish direct contact with the tissue (substrate) and become fixed to the matrix components to begin their migration and implant themselves in target organs (Figure 2).

Cell migration is a vital process that is altered in various chronic diseases increasing their severity; in this process, the cytoskeleton and associated motor proteins play a crucial role [5,42,43]. Some of these proteins are expressed in specific cell types and under pathological circumstances their expression is modified. During migration, a cell polarizes and assembles contractile structures such as the actin and myosin filaments, these filaments participate in the cellular propulsion [42,44]. The direction is controlled by the gradient of chemotactic stimuli and by the interplay of different players such as chemokines, adhesion molecules, motor, and structural proteins of the cytoskeleton [44–48]. A key signaling axis in the migration, infiltration and engraftment of B-ALL cell into testis [49] and across Choroid Plexus epithelium in the CNS [50] is the chemokine CXCL12 and its corresponding receptor CXCR4 that activate the small GTPase Rac1 that remodels actin to promote the migration. Another key player in ALL migration is alpha-6 integrin, part of the Laminin receptor [51], that is expressed in samples of primary B and T ALL patients and its expression increases in residual disease after chemotherapy [52–54]. All the ligands and their receptors, when are overexpressed in stromal cells, provide an optimal environment for the release of intracellular signals that enhance the recruitment of cells to the tissue, although, the specific chemokines, receptors, and particularly the motor proteins involved in these migratory responses of leukemia cells are not fully known [55–57].

This review focuses on the involvement of class I Myosins with cancer, in particular we will discuss the possible mechanisms by which myosins could contribute to cancer development and progression, particularly how Myo1g might enhance cellular motility and how this motility may promote the formation of infiltrates and increase the risk in diagnosed patients with ALL.

### Class I Myosins molecular motors in cell migration and cancer

Most of class I Myosins are involved in control of membrane tension and surface remodeling, It has been shown that a decrease in class I myosin expression reduces membrane tension, while overexpression increases it [6,14,20,58,59]. This has consequences in the form and size of F-actin-based structures that contribute to the membrane topology and cell migration.

Class I myosins alter cell migration. Specifically, it is known that myosin 1a, 1b, 1 c, 1 g, and 1e regulate the motility of diverse cellular lineages. Myo1b and Myo1c are the best characterized class I myosins involved in cell migration and emergent evidence highlight their role in different types of cancer.

**Myosin 1a** is implicated in *Dyctiostelium* cell migration but so far there are no studies in mouse or human cells [60–62]. Myo1a control different aspects of the intestinal microvilli, like the correct localization of proteins at the brush border [63–65], and the loss of Myo1a could contribute to increased Epithelial Mesenchymal Transition (EMT) [3] and recently Mazzolini et al demonstrated that Myo1a may function as a tumor suppressor in colon cancer, they found that the absence of Myo1a or cells expressing Myo1a mutants that generated a truncated protein resulted in partial loss of apico-basal polarity, those cells also failed to differentiate correctly, furthermore using the soft agar assay they found that Myo1A deficient or Myo1A knockdown resulted in more colonies indicating increased tumorigenicity of Myo1a deficient cells [26]. The same group also demonstrated that Myo1a, which normally is highly expressed in the gastric mucosa, is often mutated in gastric tumors [27]

**Myosin 1b** drives cellular migration during embryonic development and controls cellular propulsion through filipodia, membrane trafficking, and exocytosis [46,66,67] *in vitro* studies show that Myosin 1b prompts actin movement and abates actin growth by reducing the length of actin filaments through removal of actin monomers from the barbed end of F-actin, becoming Myosin1b in the first actin depolymerase of its kind. This activity has profound consequences in the actin architecture by changing the mesh-size, or the cortical thickness and therefore the cortical contractility [68]. Myo1b over expression has been reported in head and neck squamous cell carcinomas (HNSCC) [69]. Squamous cancer cells spread to lymph nodes this event is associated with poor prognosis. Recently, Ohmura et al demonstrated that Myo1b over expression directly regulated cancer cell proliferation and migration [70], these results indicate that Myo1b is a potential molecular marker of metastasis in HNSCC. Myosin 1b is also upregulated in Cervical Cancer (CC) where it promotes cell proliferation, migration, and invasion, and has been demonstrated that high expression of myo1b is associated with poor survival of CC patients. Although the cause of myo1b overexpression is unknown, its effects on CC are due at least in part to activation of Metalloproteinases-1/9 (MMP1/9) [29]. Myo1b is also overexpressed in Esophageal Squamous Cell Carcinoma (ESCC) clinical samples, in this cancer Myo1b is associated with cancer cell aggressiveness and metastasis, downstream genes controlled by Myo1b includes MMP1/13 both of which are highly expressed in ESCC clinical specimens [71]. Myo1b is overexpressed in the highly metastatic prostate cancer cell line PC-3, where it might increase cortical tension, granting cells to migrate through tight extracellular matrices *in vivo*, pointing its key role in 3D migration [72]. A recent study indicates that Myo1b is involved in gliomagenesis, here the authors found that the splicing factor SRF1 promoted the over expression of a longer isoform of Myo1b that localized to membrane and showed oncogenic activity [73]. Finally, Xie. et al reported that Myo1b is overexpressed in colon cancer patients, here Myo1b increased the migration and invasion of colon cancer cells both *in vitro* and *in vivo* through a mechanism that included activation of RhoA and cytoskeletal remodeling [74], those results suggest that Myo1b could be a useful biomarker of high potential of metastasis and poor prognosis in colon cancer and potentially could be used as a therapeutic target for colorectal cancer metastasis

**Myosin 1 c** was the first myosin described in the nucleus [75] and recently the participation of nuclear myosin in transcription, DNA repair, and chromosome organization has been extensively reviewed [76–79] and references therein. Myo1c participate in the transport G-Actin to stabilize lamellipodia and filopodia, also controls the recycling of Lipid Raft membranes and associated proteins that are important for cell spreading and migration [59,80–83]. Myo1c is overexpressed in gastric cancer [84]; a recent study found that patients with overexpression of miRNA 137 showed a downregulation of Myosin 1 c and higher survival rates, Myosin 1 c has three isoforms (A, B, and C), Isoform A was the target of miR137, interestingly the same isoform A is overexpressed in prostate cancer, which might make it a potential molecular marker for prostate cancer detection [85]. Recently, Saidova et al confirmed that overexpression of Myo1c isoform A is a specific marker for both prostate cancer cells and prostate cancer cell lines [86]. An analysis of Myosin 1 c expression in a series of endometrial carcinomas showed a direct correlation between the carcinoma stage and reduced expression levels of Myosin 1 c, and in vitro reduction of Myo1c in cell lines by siRNA resulted in reduced cell migration and adhesion. All these results suggest that Myosin 1 c could act as a tumor suppressor [87]. Another line of evidence suggest that Myosin 1 c is involved in angiogenesis, a key event for the growth of new blood vessels required for tumors to grow, Myo1c participates in Vascular Endothelial Growth Factor (VEGF)-induced endothelial cell proliferation and migration by controlling signaling events downstream of VEGFR2 such as VEGFR2, c-Src, and mitogen-activated protein kinase/extracellular signal-regulated kinase-1/2 (ERK1/2) phosphorylation [88]. Myo1c isoform B is a key player in estrogen-responsive breast cancer since it brings about assembly of estrogen receptors involved in cell proliferation and gene transcription [89,90]. Further in vitro evidence showed that the SH3 domain Binding Glutamic acid Rich Like 3 (SH3BGRL3) protein, which is broadly expressed in human cells and overexpressed in tumors, modulates cytoskeletal activities through calcium-dependent interaction with Myo1c, remarkably increasing cell migration of MDA-MB-231 breast cancer cell line [91].

**Myosin 1d** is broadly expressed, being the brain and the nervous system the places of highest expression for this short-tailed myosin [92], this localization implies that Myo1d is a player for nervous system development and neurological conditions. In contrast to other class I Myosins, Myo1d exhibits reduced actin-activated ATPase in the presence of Calcium, an indication of control differences of class I myosins [93], Myo1d overexpression contributes to colorectal and breast carcinogenesis by upregulating Receptor Tyrosine Kinases (RTKs) levels. To do this, Myo1d attaches RTKs of the EGFR family (except ErbB3) to the cytoskeleton, this interaction involves the direct binding of the Myosin 1d TH1 Tail domain and the kinase domain in the RTKs resulting in stabilization at the plasma membrane and increased downstream signaling [94]. Myo1d is also overexpressed in prostate cancer, however, so far there is no direct evidence of the function of Myo1d on those cells [95].

**Myosin 1e** is broadly expressed in humans and it is found in the highest levels in kidney, prostate, colon, liver, and ovary [96], Myo1e shuttles to active lamellipodia and sites of active actin polymerization to transport molecules involved in actin growth, also moves near early adhesion sites where it deposits proteins important for adhesion stabilization [97]. Myo1e is highly expressed in podocytes and is related to cellular motility by regulating membrane-cytoskeleton interaction in kidney cells, regulates lamellipodia formation [97,98]. Myo1e is involved in receptor mediated endocytosis [99,100] and recently was described as an essential component in invadosomes [101], these structures are important to degrade extracellular matrix and promote cell migration and invasion, the same group reported that Myo1e promotes tumor progression and metastasis in breast cancer by induction of proliferation and tumor cell de-differentiation [28]. Recently, Myo1e has been identified as a partner of Leukocyte-Specific Protein 1 (LSP1) and this bimolecular complex regulate events associated with actin cytoskeleton remodeling in macrophages such as phagocytosis, lamellipodia formation, focal adhesion dynamics, and cell migration [102], the interaction of Myo1e with LSP1 suggests key roles during immune responses mediated by macrophages. Another evidence of the role of Myo1e in cell migration were shown in Myo1e-deficient neutrophils that display Intermittent Rolling, as a consequence the neutrophils fail to transmigrate due to interchange of rolling and jumping among other events involved in neutrophil extravasation [103]. Finally, together with other genes, high expression of Myo1e correlates with poor prognosis of Basal Like Breast Cancer patients [104], since Myo1e is a key component of invadosomes a possibility is that Myo1e acts as a tumor promoter and a marker of invasive tumors [28].

**Myosin 1 f** is the other long-tailed myosin I, and in neutrophils regulates nuclear shape changes during migration in 3D environments and defects in Myo1f function alter extravasation of neutrophils to sites of inflammation and prevent the corresponding immunological response [105,106]. Myo1f mutations were reported recently in patients with thyroid cancer, and upon over expression of the mutant Myo1f the authors found altered mitochondria dynamics, increased mitochondrial mass and elevated Reactive Oxygen Species (ROS) production, furthermore they found that the mutation conferred significant advantage in colony formation, invasion and anchorage-independent cell growth [107], Myo1f is described as one of the fusion partners of MLL in Acute Myeloid Leukemia [108,109], so far is unknown functional significance of this fusion, however fusion with MLL is often correlated with cell transformation and Leukemogenesis [110,111]. Interestingly Myo1f has been detected as a fusion with VAV1 (a Guanine Nucleotide Exchange Factor, GEF) in Peripheral T Cell Lymphomas (PTCL), a highly aggressive hematopoietic cancer of poor prognosis [112,113], in PTCL probably is VAV1 who promotes most of the transformation events, however, further studies are necessary to explore those possibilities.

**Figure 3.**
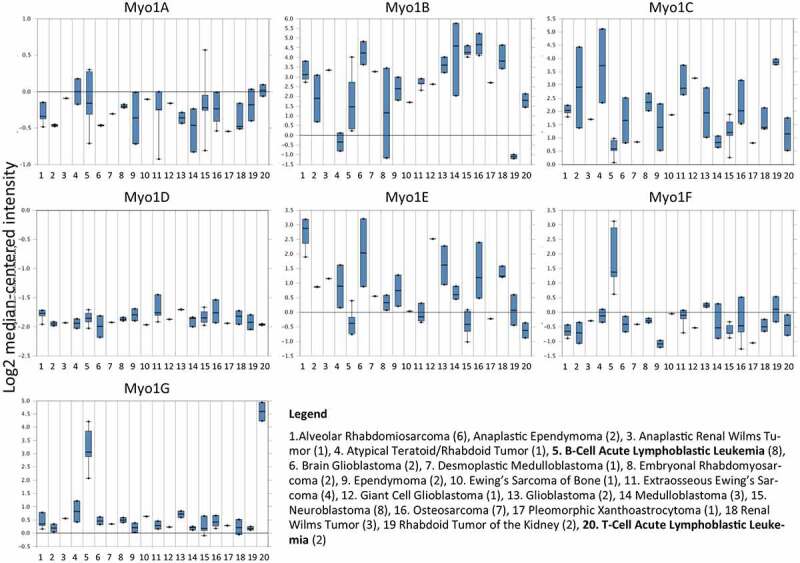
**Class I myosin expression in 20 different cancer types**, data from Oncomine, note differential expression of the class I Myosins, in lanes 5 and 20 corresponding to Acute Lymphoblastic Leukemia from B (5) and T (20) Cell origin, compared with the rest of different cancer types. Myo1h is not present in the database.

**Myosin 1 g** is expressed in hematopoietic cells and originally was identified as the human minor histocompatibility antigen HA-2 [114,115] Myo1g is enriched in microvilli, key structures involved in the binding to vascular endothelium, of B-lymphocytes and T-lymphocytes and other hematopoietic cells [23,25,116,117]. We reported that Myosin 1 g deficient B-lymphocytes display shorter trajectories, partial impairment in cell polarization and slower cell migration, presumably as a consequence of reduced membrane tension [23,118]. Surprisingly, Myo1g deficiency in T-lymphocytes resulted in increased cellular speed, but reduced the turning angle when cells migrated or move around the antigen-presenting cell under limited amounts of antigen [20]. The difference in the response between B-cells and T-cells with Myosin 1 g deficiency is not easily explained, one possibility is their unique migration pattern. Although both cell types display the amoeboid migration mechanism [119–121], which is a mode of fast motility driven by short-lived, and relatively weak interactions. Cells displaying this migration phenotype are deformable and show high motility speeds due to the lack of focal contacts, this migration mode is well characterized in neutrophils and T cells; however, B-lymphocyte migration mechanism is in general slower and only recently is been characterized *in vitro* [122,123]. Another important feature of Myosin 1 g is that upregulates trafficking of adhesion molecules such as CD44, controls lipid raft formation, exocytosis of integrins and Myosin 1 g forms a complex with signaling molecules such as RalA and its GEF to modulate spreading and migration [58,118]. So far there are no studies linking myo1g to Cancer, however, we interrogated Oncomine [124], a repository for expression studies and found that myo1g is overexpressed in Leukemia, this expression seems to be specific since myo1c one of the more widely expressed myosins is downregulated in leukemia and over expressed in other tumors (Figure 3). Those results prompted us to evaluate the expression of myo1g in a cohort of patients with leukemia, we found that there is an increased expression in those patients, especially in high risk-patients, currently we are evaluating if this expression correlate with aggressiveness or other clinical manifestations [125]. As we hypothesized earlier (Figure 2), our proposal is that B lymphocytes overexpressing myo1g are intrinsically more apt to migrate and that this ability together with increased cell contacts with the vessels allow the cells to migrate more efficiently to tissues where normally they are not supposed to migrate, producing in this way more infiltrates to the CNS and testis, which are strong indicators of severity of the disease in Leukemia. Studies under way will evaluate the ability of B cells over expressing myo1g to migrate compared to cells expressing low levels of this protein, with those studies we will try to correlate high expression of Myo1g with clinical outcomes in patients with ALL.

So far **Myosin 1 h** has not been related to cancer will not be discussed further.

## Conclusions

In summary, there is increasing evidence that changes in class I myosin expression could contribute to changes in cell migration and metastasis in different cancer cells. Class I myosins play roles in different cellular processes, such as intracellular transport, formation of cell surface projections, regulation of endocytosis, exocytosis and phagocytosis and importantly in membrane tension and cell migration and recently has been shown that they could contribute to cancer initiation and progression by different mechanisms, some of them as tumor suppressors, others increasing cancer cell motility and consequently metastasis, as we proposed for Myosin 1 g, and others favoring cell proliferation and avoiding differentiation. However, these motor proteins only recently have been the focus of interest in cancer and undoubtedly, with further research a more complete and detailed landscape of how Myosin 1 g provides B-ALL cells the capability to infiltrate CNS and testis will be developed. In this context, one first approach to assess the contribution of upregulated Myosin 1 g is by evaluating the molecular interactions with the barriers in CNS and testis and migration mechanism either using B-ALL cells from patients or B cell lines overexpressing Myosin 1 g in human-derived in vitro models that recapitulate the crossing through barriers of CNS and testis. We anticipate that knowledge obtained from this and other approaches will shed light about Myosin 1 g and its potential as a reliable biomarker or therapeutic target for Leukemia.
